# Porcelain Inlays

**Published:** 1903-04

**Authors:** T. Victor Smith

**Affiliations:** Philadelphia


### PORCELAIN INLAYS.[1]

BY DR. T. VICTOR SMITH, PHILADELPHIA.

Porcelain inlays are daily becoming more in demand, and it is not an uncommon thing for a new patient to ask, "Do you put in the new kind of filling?" To dentists it is not altogether new, but there is constant improvement and progress in the manufacture of materials and the methods of using them. Nearly all dental colleges have incorporated practical as well as didactic instruction in their curriculum, and one State board, I believe, demands a porcelain inlay as one of its requirements. Hence the age is one of progress, and it behooves the young practitioners, as well as the old, to keep well informed, especially regarding the latest principles of performing operations in porcelain.

I will endeavor to give you some suggestions as I have gathered them from various journals and other sources of information and from personal experience, without going into very much detail, as I take it for granted that you all have a knowledge of the general principles in porcelain inlay operations.

In the first place, I would like to mention a word in regard to cavity formation; most students are prone to make their cavities too shallow. We are not allowed the use of undercuts, of course, but it is necessary to make use of all the retention possible, especially in contour operations extending to the occlusal surfaces. In simple cavities on labial and approximal surfaces, not involving the pulp, it is best to make the floor of the cavity as

---

[1] Read before the Academy of Stomatology, Philadelphia, October 28, 1902. Discussed on page 201.

flat, and the side walls as perpendicular therefrom, as is possible, always, of course, considering the direction in which the matrix is to be removed. The same principle could be applied to many approximo-occlusal cavities that do not have too strong a bite and are not extensive.

Where the pulp has been exposed and cannot be capped to advantage, it is best to devitalize and use the pulp-chamber for the bulk of the body, thereby giving additional cement surface and mass to the filling to withstand the force of occlusion. A very good method, when the bite is close and the operation extensive, is to utilize the pulp-canal for anchorage by inserting a pin into the inlay which is to extend into the canal. This is done by first fitting the pin, a piece of platinum iridium wire, about 19 or 20 standard gauge, into the canal and clipping to the desired length. This is then removed and the platinum-foil adjusted and burnished to fit, the pin reinserted through the rupture in the floor of the matrix, and an application of foundation body mixed with a thin solution of gum tragacanth is flowed around the pin. This is carefully dried with warm air and the whole gradually teased from the cavity. It is then baked, more foundation body is added, but not enough to overrun the edges, and then baked again. It is now ready to be tried in the cavity and have the platinum burnished thoroughly. Over the margins, then, the enamel bodies can be placed and baked to suit. Special care must be taken not to heat or cool too rapidly in baking because of the uneven expansion and contraction of the platinum and porcelain.

A somewhat similar procedure is a unique way of repairing fractured incisors, not involving the pulp, by drilling a supplemental cavity in the palatine surface, making the matrix and a pin to fit through it into the supplemental cavity. All is removed and baked to completion, and when reinserted the cement flowing round the pin is scraped away and its space eventually filled with gold, thus giving a secure and insoluble anchorage. The pin may be made of an ordinary plate tooth pin ground flat on one side to approximate the pulpal wall of the palatine or lingual extension.

The first case that I treated in this way was about a year ago. A young man, a "cow puncher" he called himself, came into my office with the superior right central incisor fractured mesio-distally about midway between the cervix and incisal edge, I should judge. His bite was strong. The pulp fortunately had receded to a considerable extent, due to neglect in having the tooth cared for. I knew instantly that an inlay would not remain in position with the ordinary anchorage obtainable, so resorted to the pin and supplemental cavity. The inlay still is in place, and is giving perfect satisfaction. Since then I have repaired several corner fractures in my private practice and in the clinic at the Dental Department, University of Pennsylvania, in the same way, with equally good results.

A few suggestions in securing a good matrix, for upon this almost the entire success as to fit and permanence of the inlay depends. In burnishing the matrix it is well to anneal the platinum several times to keep it as soft as possible. Place it over the cavity orifice and outline the edges, then with a ball burnisher gradually draw from the edge to the centre. When well in the cavity a piece of spunk is placed at the cervical portion and thrust home, another piece added, and so on, until the entire cavity is full, thereby swedging closely to all walls and angles. An instrument can then be held on the spunk and another used by the operator to burnish the edges more carefully. The spunk is then removed piece by piece in advance of the matrix.

The next problem is building in the porcelain body and shading it. My experience has been largely with the Brewster bodies, and although there are several other very good ones on the market, Brewster's has given me the best results. I prefer having it a little finer than it is manufactured, as it gives a more compact and smooth appearance. The palatine portion of the matrix is filled with the foundation body, usually of a deeper yellow than the proper shade of the tooth. Care should be taken to allow the body to extend only to that portion of the matrix representing the margin of the cavity. It is now baked, and the platinum trimmed and replaced in the cavity. The shrinkage will allow a very careful and final burnish around the margin. If there is any spring to the platinum at this point, heat again to redness in the furness, replace in the cavity, and stretch a small piece of rubber dam over the entire filling; then burnish over the rubber dam. The shading is very cleverly accomplished by Dr. Reeves, of Chicago, by using darker colors, or lighter ones, in larger bulk and fusing over these an enamel general in color to all parts of the tooth. For instance, suppose we desired to make an inlay that would be quite yellow at the curve and of a bluish tinge at the occlusive. Rather a deep shade of yellow would be applied at the cervical portion of the inlay, and a decided blue at the incisal portion. This would be fused. Over both of these would be applied a light yellow, which would modify the other shades.

If there were still remaining any portion of the inlay with insufficient contour, a new transparent body or enamel would be used to restore the proper shape. This new body is called "XX," and was prepared by Mr. Brewster for Dr. Reeves for just such occasions. It fuses slightly below the temperature required to fuse the regular enamel body. Dr. Reeves claims for his method avoidance of shadow, translucency, and prevention of cement reflection from underneath.

The finished inlay should fit the cavity so closely that only the thinnest layer of cement is present after the inlay is inserted. The retention is best effected by simply etching the cavial surface of the inlay with concentrated hydrofluoric acid. To do this place the inlay face down on a piece of paraffin or wax and with a warm spatula draw to the edges, cool, and apply acid with a hard rubber point for a minute or two. After the inlay has been inserted scrape away excess cement and varnish with sandarach. Then apply paraffin, which will adhere for a much longer time to the sandarach than to the surface of inlay or tooth. Never have the rubber dam over the tooth when trying to match a shade, because when dry teeth are several shades lighter.

Excellent results may be obtained by the use of porcelain enamel paints, both by fusing on the surface of an inlay and under another modifying shade of enamel body.

